# Berberine and Berberine-Containing Alkaloid Preparations in Preclinical Models of Endometriosis and Adenomyosis: A Systematic Review

**DOI:** 10.3390/nu18213647

**Published:** 2026-11-02

**Authors:** Rafał Watrowski, Stoyan Kostov, Mario Palumbo, Sebastian D. Schäfer, Ingolf Juhasz-Böss, Radmila Sparić, Angel Yordanov, Salvatore Giovanni Vitale, Ibrahim Alkatout

**Affiliations:** 1 Department of Gynecology, Helios Hospital Müllheim, Teaching Hospital of the University of Freiburg, 79379 Müllheim, Germany; 2 Faculty of Medicine, University of Freiburg, 79106 Freiburg, Germany; 3 Department of Gynecology, Hospital “Saint Anna”, 9002 Varna, Bulgaria; drstoqn.kostov@gmail.com; 4 Research Institute, Medical University Pleven, 5800 Pleven, Bulgaria; 5 Department of Public Health, School of Medicine, University of Naples Federico II, 80138 Naples, Italy; mario.palumbo@unina.it; 6 Department of Gynecology and Obstetrics, Clemenshospital Münster, 48153 Münster, Germany; seb.schaefer@alexianer.de; 7 Department of Obstetrics and Gynecology, Medical Center—University Hospital Freiburg, 79106 Freiburg, Germany; ingolf.juhasz-boess@uniklinik-freiburg.de; 8 Faculty of Medicine, University of Belgrade, 11000 Belgrade, Serbia; radmila@rcub.bg.ac.rs; 9 Clinic for Gynecology and Obstetrics, University Clinical Centre of Serbia, 11000 Belgrade, Serbia; 10 Department of Gynecologic Oncology, Medical University Pleven, 5800 Pleven, Bulgaria; angel.jordanov@gmail.com; 11 Obstetrics and Gynecology Unit, “Gaspare Rodolico-San Marco” University Hospital, Department of General Surgery and Medical Surgical Specialties, University of Catania, 95124 Catania, Italy; salvatoreg.vitale@unict.it; 12 Department of Obstetrics and Gynecology, Medical University of Innsbruck, 6020 Innsbruck, Austria; ibrahim.alkatout@i-med.ac.at

**Keywords:** berberine, endometriosis, adenomyosis, supplements, diet, microbiome, estrogen, progesterone, GLP-1, inflammation, preclinical evidence

## Abstract

**Background/Objectives:** Berberine is increasingly promoted through social media and endometriosis-related websites as a non-hormonal supplement, although its disease-specific evidence remains unclear. This PRISMA-compliant systematic review evaluated experimental and clinical evidence for berberine in endometriosis and adenomyosis. **Methods:** PubMed/MEDLINE, EBSCOhost, BASE, CNKI Overseas, CENTRAL, and Google Scholar were searched from inception to 3 August 2026, supplemented by targeted manual searches. Clinical studies and experimental studies using endometriosis- or adenomyosis-related cellular or animal models were eligible. Purified berberine formed the primary evidence set. Chemically characterized multi-alkaloid preparations containing berberine were analyzed separately. Risk of bias was assessed with SYRCLE for animal studies and a predefined domain-based appraisal for in vitro studies. A structured qualitative assessment examined confidence in the synthesized findings. Contextual evidence from outside endometriosis and adenomyosis concerning steroid signaling, gut microbiota, microbial metabolites, and GLP-1 was discussed separately. **Results:** Six preclinical studies were included: three on endometriosis and three on adenomyosis, comprising four in vitro and two animal studies. Five investigated purified berberine and one a protoberberine-rich multi-alkaloid fraction. No clinical study was identified. Berberine reduced cell proliferation or viability and, where directly assessed, migration and invasion. It also reduced inflammatory and angiogenesis-related signaling, and, in an adenomyosis mouse model, myometrial infiltration and hyperalgesia. Reduced proliferation or viability recurred in all four cellular studies, with lower IL-6 and VEGF reported across cellular and animal adenomyosis models. Reported molecular effects involved NF-κB, STAT3, TLR4-associated signaling, VEGF, MMPs, TRPV1, COX-2, miR-429, and lncRNA H19. Gut microbiota modulation, short-chain fatty acids, intestinal barrier function, estrogen metabolism, GPER1, and GLP-1/GLP1R signaling remain plausible indirect routes that have not been demonstrated in disease-specific berberine studies. **Conclusions:** Berberine shows disease-relevant activity across a small and heterogeneous preclinical evidence base. Clinical efficacy, clinically achievable exposure, reproductive safety, and microbiota- or hormone-mediated effects remain unestablished.

## 1. Introduction

Endometriosis and adenomyosis are related conditions characterized by ectopic growth of endometrium-like tissue (adenomyosis–in myometrium, endometriosis–in all other extrauterine localizations) [1,2]. Adenomyosis and endometriosis coexist in up to 80–90% of cases [3]. Both originate from altered eutopic endometrium and share several pathophysiological features, including genetic and epigenetic alterations, local estrogen excess and progesterone resistance, inflammatory and immune dysregulation, enhanced proliferation and invasion, angiogenesis, and fibrosis [2,4,5,6]. None of the classical theories can sufficiently explain both diseases [5,7], so the “seed-and-soil” metaphor appears to best describe the sum of underlying pathophysiological processes [8]. Endometriosis affects at least 10% of women worldwide [1]. The exact prevalence of adenomyosis is unclear, with estimates ranging between 9 and 62% [9]. The true prevalence of both diseases remains occult, as the traditional surgical-histological “gold standard” is invasive and not practicable for screening purposes [10], ultrasound diagnosis is limited by lesion localization and examiner skills [2,11,12], whereas upcoming non-invasive methods utilizing saliva, menstrual blood, urine, or serum samples [8,13,14,15,16] are still preliminary, incompletely standardized, and not routinely integrated into diagnostic pathways.

Common manifestations of adenomyosis and endometriosis include dysmenorrhea, chronic pelvic pain, dyspareunia, and subfertility, with abnormal uterine bleeding and recurrent pregnancy loss additionally associated with adenomyosis [1,2], and multifaceted systemic manifestations in endometriosis, such as bowel or urinary symptoms (“visceral syndrome of endometriosis” [17] or “endo belly” [18]), cardiovascular and autoimmune comorbidities, and psychological manifestations, such as fatigue, depression, anxiety, attention-deficit hyperactivity disorder and sleep disturbances [19,20]. Pain severity is only weakly related to lesion number or anatomical extent. Peripheral sensitization, neuroinflammation, pelvic floor dysfunction, and central pain processing also contribute to the clinical pain phenotype [21,22,23]. The burden may affect sexual health, education, employment, and long-term healthcare use [1,24]. The complexity and severity of manifestations and consequences have shifted the perception of endometriosis from a gynecologic-only to a systemic disease [6,25].

The biology of endometriosis and adenomyosis involves altered properties of both the eutopic endometrium and the ectopic tissue. In endometriosis, ectopic cells show altered adhesion and proliferation, disturbed steroid metabolism, reduced susceptibility to cell death, and abnormal interactions with immune and stromal cells [6,26]. Local estradiol production and reduced responsiveness to progesterone favor lesion persistence. Activated macrophages, inflammatory cytokines, prostaglandins, vascular growth factors, reactive oxygen species, and extracellular matrix remodeling further contribute to inflammation, angiogenesis, fibrosis, and pain [1,24,26]. Adenomyosis shares many of these alterations. Lesion development within the myometrium is additionally associated with junctional-zone disruption, uterine hyperperistalsis, repeated tissue injury and repair, epithelial–mesenchymal transition, neuroangiogenesis, and fibrosis [2,5]. The relative contribution of these processes differs between superficial peritoneal, ovarian, and deep infiltrating endometriosis, as well as between adenomyosis phenotypes. It may also depend on disease duration, previous treatment, and the local tissue environment. A compound acting on several of these processes could therefore produce different effects across lesions and experimental models.

Current treatment of adenomyosis and endometriosis consists mainly of analgesia, hormonal therapy, and surgery [2,10,24,27]. Combined hormonal contraceptives, progestogens, gonadotropin-releasing hormone agonists and antagonists, and aromatase inhibitors reduce ovarian or local estrogenic stimulation through different mechanisms [24,26]. No drug is specifically approved for adenomyosis. Despite this, progestogens, the levonorgestrel-releasing intrauterine system, and gonadotropin-releasing hormone analogues are commonly used [2]. Hormonal treatment can be effective in pain relief, but one-quarter to one-third of women receiving first-line hormonal therapy report no or incomplete response [23]. Adverse effects, contraindications, recurrence after discontinuation, and incompatibility with active attempts to conceive further restrict long-term use in some patients [24]. In endometriosis, surgery can reduce pain and restore anatomy in selected cases, but it is invasive, technically demanding, operator dependent, may decrease ovarian reserve after endometrioma surgery, and—even if performed by an experienced surgeon—does not prevent recurrence [2,10]. In adenomyosis, the uterus-sparing surgical options (resection or ablative techniques) are less common as they are non-definitive and impair myometrial integrity [28,29,30].

All these limitations have increased interest in fertility-sparing, non-hormonal approaches directed at inflammation, angiogenesis, fibrosis, metabolism, immune regulation, oxidative stress, or pain processing. Among non-hormonal strategies, dietary approaches and supplements have gained particular popularity. Clinical data are available for low-FODMAP and Mediterranean-style dietary interventions and for supplementation with omega-3 fatty acids and *N*-acetylcysteine, while resveratrol, vitamins C and E, and probiotics are currently being investigated [31,32,33,34]. Social media and websites are a relevant information source for patients and users seeking information [35]. Dietary and supplementary advice spread rapidly through social media and endometriosis-related websites regardless of the strength of the underlying evidence [35].

Berberine has entered online discussions of endometriosis before its disease-specific evidence has been established. As of August 2026, more than 63,000 Instagram posts were associated with the hashtag *#berberine*, accompanied by extensive content on glucose control, body weight, gut health, hormones, and women’s health [36]. Selected endometriosis-oriented websites already list berberine among the “best supplements for endometriosis symptom relief”, attribute possible benefits to reduced lipopolysaccharide exposure, immune modulation, estrogen metabolism, the gut microbiome, endometrial receptivity, or fertility, and even discuss a commonly recommended regimen of 500 mg three times daily [37,38,39,40]. These recommendations combine findings from metabolic disorders, polycystic ovary syndrome, gastrointestinal research, adenomyosis, and isolated cellular models. No clinical study has evaluated berberine as a treatment for endometriosis or adenomyosis.

Berberine is a yellow, bitter, quaternary protoberberine isoquinoline alkaloid occurring in medicinal plants of the *Coptis*, *Berberis*, and *Phellodendron* genera; hydrochloride and sulfate are commonly used salt forms [41]. Berberine-containing preparations have long been used in Chinese and other traditional medicines, particularly for gastrointestinal infections and diarrhea, while modern clinical research has focused mainly on metabolic and gastrointestinal disorders [42]. Oral bioavailability of the parent compound is very low (<1%), owing to limited intestinal absorption, transporter-mediated efflux, and extensive first-pass metabolism. Phase-I metabolites undergo further glucuronide or sulfate conjugation, while substantial intestinal exposure permits bidirectional interactions with the gut microbiota [41]. Gut bacteria can convert berberine to the more readily absorbed dihydroberberine, whereas berberine itself alters microbial composition and metabolic activity [43,44]. Reported effects involve AMPK, NF-κB, PI3K/AKT, MAPK, mitochondrial and redox regulation, epithelial barrier function, cytokine signaling, and cell-cycle control, with direction and magnitude varying considerably by model, dose, and exposure conditions [41,45].

Experimental studies in female reproductive tissues showed that berberine inhibited estradiol- and progesterone-stimulated proliferation and induced cell-cycle arrest in human leiomyoma cells, while normal myometrial cells were less affected at the same concentrations [46]. Direct interaction with estrogen or progesterone receptors was not examined. In ovariectomized rats, berberine altered gut microbial composition and increased equol, a microbiota-derived isoflavone metabolite with preferential affinity for estrogen receptor β, without restoring circulating estradiol or progesterone; these effects were absent or markedly reduced in germ-free animals [47]. In a rat model of acute uterine inflammation, oral berberine reduced cytokine expression, oxidative injury, and TLR2/PI3K/AKT activity while protecting uterine tissue from inflammation-associated apoptosis [48]. Its effects on cell survival therefore appear context-dependent. Possible interactions with estrogen and progesterone receptors, microbial estrogen metabolism, and downstream steroid-responsive pathways require disease-specific investigation.

Several effects reported for berberine outside endometriosis and adenomyosis overlap with processes relevant to both diseases, including regulation of cell proliferation and survival, inflammatory and redox signaling, steroid-responsive pathways, intestinal barrier function, microbial metabolism, and endogenous GLP-1 secretion [41,43,45,46,47,49]. Evidence for these effects comes from different experimental settings, including uterine, intestinal, metabolic, and estrogen-deficiency models, and does not establish that they also occur in endometriosis or adenomyosis. Their possible relevance is supported by independent evidence implicating the same biological processes in these diseases [4,50,51].

Based on these findings and theories, we for the first time, systematically identified and evaluated all eligible preclinical and clinical studies of berberine in endometriosis and adenomyosis. The primary objectives were to characterize the experimental models, interventions, outcomes, and mechanisms directly examined in these studies and to assess their methodological and translational limitations. The secondary objectives were to evaluate biological and pharmacological links supported outside endometriosis and adenomyosis and to classify them according to their proximity to the disease-specific evidence. Particular attention was given to interactions with estrogen and progesterone signaling, the gut microbiota–barrier–immune axis, microbiota-derived metabolites, and GLP-1-related pathways.

## 2. Materials and Methods

### 2.1. Protocol Registration and Reporting Standard

This systematic review was prospectively registered with the Open Science Framework (OSF) on 18 July 2026 (registration DOI: 10.17605/OSF.IO/WCXR8; associated project: https://osf.io/rf6aq, accessed on 30 October 2026). The review was conducted and reported in accordance with the Preferred Reporting Items for Systematic Reviews and Meta-Analyses (PRISMA) 2020 statement [52]. The completed PRISMA 2020 checklist is provided as Supplementary Table S1.

### 2.2. Eligibility Criteria

Original studies were eligible if they investigated berberine in endometriosis or adenomyosis. Eligible designs included human clinical studies, animal models, primary patient-derived cells, organoids, tissue explants, and established cell lines explicitly used to investigate either disease. Studies using normal endometrial or myometrial cells without a defined disease-related experimental context were excluded. Reviews and other publications without original experimental or clinical data were excluded.

The principal intervention was purified berberine administered as the experimental treatment, irrespective of salt form, formulation, concentration, dose, route, or duration. Studies of chemically characterized multi-alkaloid preparations containing berberine were retained as indirect evidence and analyzed separately, without attributing their effects specifically to berberine. Studies investigating related protoberberine or isoquinoline alkaloids, including oxyberberine, tetrahydropalmatine or levo-tetrahydropalmatine, without administration of berberine itself were excluded.

Outcomes of interest included lesion-related findings; cell viability, proliferation, cell-cycle distribution and apoptosis; migration and invasion; inflammatory, angiogenic and matrix-remodeling markers; nociceptive outcomes; non-coding RNAs and intracellular signaling; metabolic and metabolomic findings; pharmacokinetic data; and toxicity or safety outcomes.

### 2.3. Information Sources and Search Strategy

A systematic literature search was conducted in PubMed/MEDLINE, the EBSCOhost platform, and the Bielefeld Academic Search Engine (BASE), the China National Knowledge Infrastructure platform (CNKI Overseas), and the Cochrane Central Register of Controlled Trials (CENTRAL), covering each source from inception to 3 August 2026. EBSCOhost included Academic Search Premier, APA PsycArticles, APA PsycInfo, CINAHL, and MEDLINE. Google Scholar was searched separately for additional records.

PubMed/MEDLINE was searched using controlled vocabulary and free-text terms for berberine combined with terms for endometriosis and adenomyosis: (“Berberine”[Mesh] OR berberin*[Title/Abstract] OR “berberine chloride”[Title/Abstract] OR “berberine hydrochloride”[Title/Abstract] OR BBR[Title/Abstract]) AND (“Endometriosis”[Mesh] OR “Adenomyosis”[Mesh] OR endometriosis[Title/Abstract] OR endometriotic[Title/Abstract] OR endometrioma*[Title/Abstract] OR adenomyosis[Title/Abstract] OR adenomyotic[Title/Abstract]). The search retrieved six records.

The EBSCOhost search used: (“Berberine” OR berberin* OR “berberine chloride” OR “berberine hydrochloride”) AND (endometriosis OR endometriotic OR endometrioma* OR adenomyosis OR adenomyotic). The EBSCOhost search retrieved 13 records.

BASE was searched across all fields using: berberin* AND (endometrio* OR adenomyo*). The BASE search retrieved 12 records.

Google Scholar was searched using: “berberine” AND (“endometrio*” OR “adenomyo*”). The search returned 82 results, all of which were screened. It identified one additional eligible study, Gao et al. [53], which had not been retrieved through PubMed/MEDLINE, EBSCOhost, or BASE.

CNKI Overseas, accessed through its English-language interface, was searched in the Title/Keyword/Abstract field using *berberine AND (endometriosis OR adenomyosis)*. The search retrieved 10 records.

CENTRAL was searched using *berberine AND (endometriosis OR adenomyosis)* in the Title/Abstract/Keyword field. The search retrieved no records (n = 0).

No restrictions on publication date, language, species, study design, or publication status were applied. Search terms for related protoberberine or isoquinoline alkaloids were not added unless the record explicitly referred to berberine.

Reference lists of the included studies and relevant reviews were screened for further eligible reports. This did not identify any additional studies.

### 2.4. Study Selection

The PubMed/MEDLINE search identified six records, EBSCOhost 13, BASE 12, CNKI Overseas 10, Google Scholar 82, and CENTRAL none, giving 123 records in total. Among the 31 records identified through PubMed/MEDLINE, EBSCOhost, and BASE, 11 duplicates were removed. The remaining 20 records underwent title and abstract screening, and 13 were excluded as unrelated to the review question. All 82 Google Scholar records were screened by title, identifying one additional report for full-text assessment [53]. Of the 10 CNKI records, nine were excluded by title and one after abstract assessment because it evaluated oxyberberine without a berberine treatment arm [54]. Eight reports were therefore assessed in full text. Two were excluded because they evaluated tetrahydropalmatine or levo-tetrahydropalmatine, related protoberberine alkaloids, without administration of berberine [55,56]. Six studies met the eligibility criteria and were included in the final synthesis.

**Figure 1:**
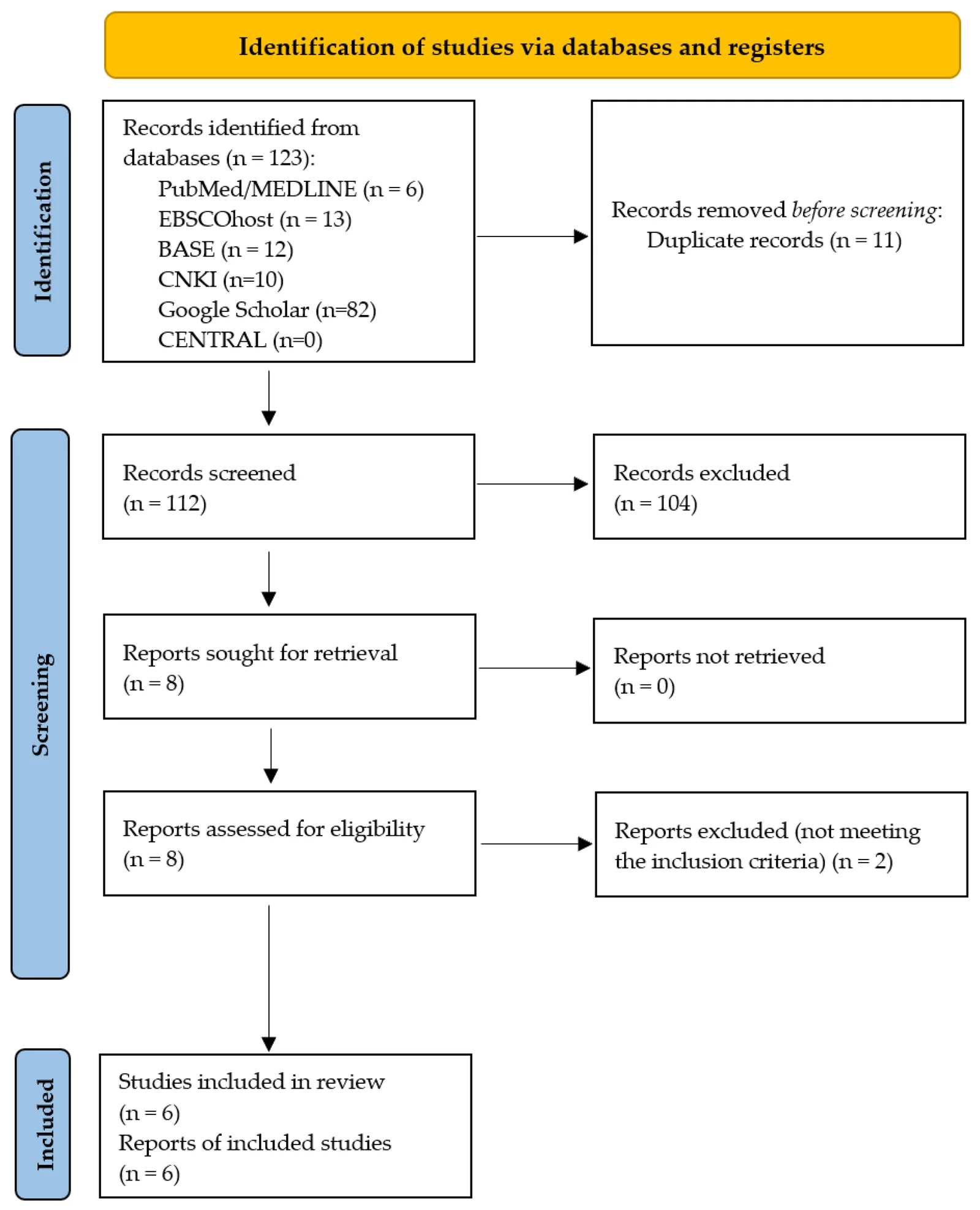
PRISMA 2020 flowchart of study identification and selection.

The study selection process is shown in the PRISMA 2020 flow diagram (Figure 1).

### 2.5. Data Extraction

RW and MP independently extracted data from the full texts of all included studies using a structured evidence table. The completed extraction was compared between the two evaluators and reviewed with SK. Discrepancies were resolved by re-examination of the original report. IA was designated to adjudicate potential disagreements, but this was not required. Extracted study characteristics included first author, year of publication, country, study design, funding, and conflicts of interest; disease investigated; experimental model; source and number of patients, animals, cell preparations, or cell lines; and biological and technical replication where reported. Intervention data included berberine identity and purity, salt or formulation, concentration or dose, route of administration, duration and timing of treatment, comparator or control condition, and co-interventions. For the protoberberine-rich preparation, the botanical source, chemical characterization, identified constituents, and available information on berberine content were recorded separately.

All reported disease-relevant outcomes were collected. These included lesion burden and histological findings; cell viability and proliferation; cell-cycle distribution and apoptosis; migration and invasion; inflammatory cytokines and chemokines; angiogenic and matrix-remodeling markers; nociceptive outcomes; non-coding RNAs; intracellular signaling; and metabolic or metabolomic findings. Pharmacokinetic measurements, toxicity, adverse effects, and information relevant to dose translation were recorded when reported. For pathway-related findings, changes in expression, phosphorylation, or localization were distinguished from experiments that directly tested functional involvement through knockdown, overexpression, pharmacological perturbation, or rescue. Information not given in the original reports was recorded as not reported and was not inferred.

### 2.6. Risk-of-Bias Assessment

Animal experiments were assessed using the Systematic Review Centre for Laboratory Animal Experimentation (SYRCLE) risk-of-bias tool [57], including sequence generation, baseline comparability, allocation concealment, random housing, blinding of caregivers and investigators, random outcome assessment, blinded outcome assessment, incomplete outcome data, selective reporting, and other sources of bias.

Because no universally accepted risk-of-bias instrument is available for in vitro studies of this type, in vitro experiments were evaluated using a predefined domain-based appraisal. The domains comprised biological relevance of the experimental model; source, identity, and characterization of cells; definition of donor number and biological replication; distinction between biological and technical replicates; adequacy of control and vehicle groups; intervention identity and purity; justification of concentration and exposure time; completeness of outcome reporting; suitability of statistical methods and experimental unit; and reproducibility of the reported findings. For pathway-related claims, additional weight was given to direct target engagement, pathway perturbation, and rescue experiments. Multi-alkaloid preparations were additionally assessed for chemical characterization and the extent to which an observed effect could be assigned to an individual constituent.

RW and SK independently performed the assessments. IA was designated to adjudicate unresolved disagreements, but no adjudication was required. Risk-of-bias judgments informed interpretation of the results and were not used as exclusion criteria.

### 2.7. Data Synthesis

The included studies differed substantially in disease model, experimental system, intervention, berberine concentration or dose, route of administration, outcome definition, and reporting format. Quantitative pooling was therefore not appropriate, and the evidence was synthesized narratively. Studies were grouped by disease (endometriosis or adenomyosis), experimental setting (in vitro or in vivo), and intervention type (purified berberine or a berberine-containing multi-alkaloid preparation). Primary patient-derived models and established cell lines were considered separately where this affected interpretation. Results were retained in the form reported by the original studies. No common effect measure was calculated, and no missing numerical results were imputed. Formal statistical subgroup analyses, sensitivity analyses, assessment of small-study effects or publication bias, and meta-analysis were not performed because of the small number and marked heterogeneity of the included studies.

Confidence in the synthesized findings was assessed qualitatively (Appendix A, Table A1). It considered risk of bias, consistency and independence of reports, directness of the model and intervention, biological plausibility, dose (exposure) relevance and translational relevance. Adaptations of GRADE have been proposed for animal intervention studies [58], but the present assessment addresses a mixed cellular and animal evidence base without claiming to apply that method. Therefore, formal GRADE categories or a numerical score were not assigned.

## 3. Results

### 3.1. Study Selection and Characteristics

Six preclinical studies published between 2017 and 2025 met the eligibility criteria and were included in the qualitative synthesis [53,59,60,61,62,63]. They are summarized in Table 1. No clinical study evaluating berberine for the treatment of endometriosis or adenomyosis was identified. Three studies addressed adenomyosis [59,60,63] and three addressed endometriosis [53,61,62]. Four studies were performed in vitro [53,59,60,61] and two in vivo [62,63]. Five studies investigated purified berberine, whereas Warowicka et al. evaluated a protoberberine-rich Chelidonium majus fraction containing berberine together with several other alkaloids [62]. Five studies originated from China and one from Poland.

Three of the four in vitro studies used primary patient-derived stromal cells. Liu et al. [59] investigated ectopic endometrial stromal cells (EESCs) obtained from three women with adenomyosis, and Liu et al. [60] compared EESCs and eutopic endometrial stromal cells (EuESCs) from three women with adenomyosis with normal endometrial stromal cells (NESCs) from three controls. Gao et al. [53] isolated ectopic endometrial stromal cells (ecESCs) from ovarian endometriotic cysts of three women. Gu and Zhou [61] used the immortalized human endometrial stromal cell line (HESC) without lesion-derived material or an experimentally induced endometriosis phenotype.

Berberine concentrations in vitro ranged from 20 to 200 µM. The two Liu studies used 50–200 µM, Gu and Zhou used 20–80 µM and selected 80 µM for most subsequent experiments, and Gao et al. used 20 µM [53,61]. The purified-berberine animal study administered 5, 10, or 20 mg/kg/day intraperitoneally for three weeks [63]. Warowicka et al. administered 1 g/kg of a protoberberine-rich fraction orally for 14 days [62]. No included study measured plasma, tissue, or lesion concentrations of berberine.

### 3.2. In Vitro Effects on Stromal Cell Proliferation and Survival

All four in vitro studies reported inhibition of stromal cell proliferation or viability after berberine exposure. The two adenomyosis studies from Liu and colleagues used primary patient-derived EESCs and showed concentration-dependent effects within the 50–200 µM range [59,60]. In the LPS-stimulated model, LPS increased EESC viability, whereas berberine attenuated this increase in a concentration- and time-dependent manner. At 200 µM for 48 h, berberine shifted the cell-cycle distribution toward G0/G1 and increased apoptosis compared with LPS stimulation alone [59]. The study measured downstream inflammatory and invasion-associated products but did not directly measure TLR4 or experimentally inhibit TLR4 signaling.

The subsequent study from the same research group examined untreated primary NESCs, EuESCs, and EESCs [60]. Berberine reduced the viability of EuESCs and EESCs, with greater inhibition at higher concentrations and longer exposures, while the growth of NESCs was described as only slightly affected. EESCs were more strongly inhibited than EuESCs at 200 µM; after 48 h, EESC viability was 52.06 ± 3.85% of control [60]. In EESCs, 100 and 200 µM berberine induced G1-phase arrest and apoptosis after 48 h. Cleaved caspase-3 increased, supporting activation of a caspase-dependent apoptotic response. These observations provide the only direct comparison between disease-associated and normal endometrial stromal cells in the included literature.

The two endometriosis studies also reported antiproliferative effects. Gu and Zhou [61] observed concentration- and time-dependent reductions in viability of CRL-2615 HESCs and selected 80 µM for subsequent experiments. Berberine reduced colony formation and EdU-positive cells and decreased Ki-67 and PCNA expression. However, the cellular endometriosis model used in [61] is based on HESCs and not on cell lines directly derived from endometriotic lesions.

Gao et al. [53] studied ecESCs isolated from ovarian endometriotic cysts from three patients. Berberine at 20 µM significantly reduced cell proliferation compared with untreated controls. An antiproliferative effect was thus also observed at 20 µM in primary endometriosis-derived stromal cells. The methods contain an internal discrepancy in treatment timing: the general treatment protocol specifies 0, 12, 24, and 48 h, whereas the CCK-8 protocol and figure report measurements at 0, 24, 48, and 72 h.

### 3.3. Migration, Invasion, Angiogenic and Inflammatory Phenotypes

Three primary cell studies and the CRL-2615 study assessed invasion-related phenotypes or molecular mediators. Liu et al. [59] found that 200 µM berberine suppressed the LPS-induced expression of IL-6, IL-8, TGF-β, EGF, VEGF, and MMP2 at the mRNA and/or protein level. The same marker panel was reduced by 100–200 µM berberine in unstimulated adenomyosis-derived EESCs in Liu et al. [60]. In the latter study, these changes were accompanied by reduced nuclear translocation of NF-κB/p65 and reduced STAT3 activation.

At 80µM, berberine reduced migration in a wound-healing assay and invasion through Matrigel in a Transwell assay, accompanied by decreased MMP2 and MMP9 expression [61]. The article abstract additionally refers to MMP4, although the principal figures and results emphasize MMP2 and MMP9. Gao et al. [53] independently observed reduced Transwell invasion in patient-derived ecESCs following 20 µM berberine.

VEGF was reduced in both adenomyosis EESC studies and in uterine tissue in the mouse adenomyosis model [59,60,63]. Direct measurements of vessel density, functional angiogenesis, or lesion perfusion were not performed, so the included evidence supports modulation of angiogenesis-associated signaling without demonstrating an anti-angiogenic effect at the tissue level.

### 3.4. Non-Coding RNA-Associated Mechanisms

Two endometriosis studies identified non-coding RNAs associated with the cellular response to berberine. Gu and Zhou [61] found that 80 µM berberine reduced miR-429 expression. Overexpression of miR-429 attenuated or reversed the effects of berberine on viability, colony formation, EdU incorporation, Ki-67, PCNA, migration, invasion, MMP2, and MMP9. This rescue design provides direct experimental evidence that miR-429 participates in the observed berberine response in CRL-2615 cells. Its relevance to lesion-derived endometriotic stromal cells was not examined.

Gao et al. [53] found a significant reduction in lncRNA H19 expression after berberine treatment of primary ecESCs. Proliferation and invasion decreased in parallel, and berberine and metformin produced the strongest effects among the four compounds investigated. H19 was measured as an associated molecular endpoint; H19 expression was not experimentally restored after berberine treatment, and no H19-directed intervention was performed within this experiment. The study therefore demonstrates an association between berberine exposure, lower H19 expression, and reduced proliferation and invasion, without establishing H19 as the mediator of the berberine effect.

### 3.5. In Vivo Disease Burden and Pain-Related Outcomes

Zhu et al. [63] provided the only in vivo study in which purified berberine was administered in an adenomyosis model. Adenomyosis was induced by neonatal tamoxifen exposure, and affected mice subsequently received 5, 10, or 20 mg/kg/day berberine intraperitoneally for three weeks. Uterine weight was significantly reduced at 10 and 20 mg/kg compared with untreated adenomyosis mice, while the reduction at 5 mg/kg was not statistically significant. Myometrial infiltration of ectopic endometrium was similarly reduced at 10 and 20 mg/kg.

Thermal nociception showed a broader dose response. Adenomyosis mice had reduced hotplate and tail-flick latencies before treatment. All three berberine doses increased endpoint latencies, with progressively larger effects at 5, 10, and 20 mg/kg; at 20 mg/kg, the reported latencies approached those of normal controls. Molecular analyses showed reduced TRPV1 and COX-2 expression in DRG tissue and reduced TRPV1, COX-2, VEGF, and oxytocin receptor expression in uterine tissue. Berberine also reduced NF-κB/p65 phosphorylation in DRG tissue. At 20 mg/kg, TNF-α, IL-6, and MCP-1 concentrations in DRG were lower than in untreated adenomyosis mice. TLR4 mRNA and protein expression decreased in both DRG and uterine tissue, with uterine immunohistochemistry showing the same direction of effect. No TLR4 inhibitor, agonist, knockout model, or rescue experiment was used to test whether suppression of TLR4 was required for the phenotypic response.

Warowicka et al. [62] investigated a surgically induced rat model of peritoneal endometriosis. The intervention was a C. majus fraction containing berberine, coptisine, dihydroberberine, stylopine, protopine, chelidonine, sanguinarine, allocryptopine, and magnoflorine. Twenty-one rats were assigned to the treatment group and received 1 g/kg of the fraction orally for 14 days following autotransplantation. Five treated animals had no endometriosis at the second laparotomy and were excluded from subsequent analyses, leaving 16 treated animals, in which the endometriotic lesions were excised. At the third laparotomy three months later, the authors reported endometriosis in 12.5% of the treated group and 61% of the untreated model group (*p* = 0.02). The reported 61% cannot be reconstructed as a rat-level proportion from the nine animals assigned to the model-control group, leaving the denominator or unit of analysis uncertain. Histologically, recurrent lesions in treated rats showed stromal atrophy with preserved endometrial glands, whereas untreated implants more closely resembled eutopic endometrium.

The same study identified 34 serum metabolites by ^1^H-NMR and reported several differences involving metabolites related to energy metabolism. The multivariate analysis provides limited support for a treatment-associated metabolic signature. The initial principal-component analysis did not separate the groups, and the OPLS-DA models failed CV-ANOVA validation for all comparisons except the untreated endometriosis versus sham comparison. Individual metabolite differences should therefore be interpreted separately from evidence for a reproducible global metabolomic effect.

### 3.6. Subgroup Analyses

#### 3.6.1. Endometriosis and Adenomyosis

The evidence was evenly divided between endometriosis and adenomyosis, with three studies in each disease. The adenomyosis subgroup contained two primary human cell studies and one purified-berberine animal study. All three implicated inflammatory signaling. The two cell studies consistently showed reductions in IL-6, IL-8, VEGF, and MMP2 [59,60], while the animal study demonstrated suppression of TLR4, NF-κB activation, TNF-α, IL-6, and MCP-1 and included a functional pain phenotype [63].

The endometriosis subgroup comprised two in vitro studies and one animal study [53,61,62]. Purified berberine was evaluated only in vitro. Both in vitro studies showed inhibition of proliferation and invasion-related behavior, with miR-429 and lncRNA H19 identified as candidate molecular mediators [53,61]. The sole in vivo endometriosis study used a multi-alkaloid C. majus fraction, preventing attribution of its lesion findings to berberine [62]. No study has therefore tested purified berberine in an in vivo endometriosis model.

#### 3.6.2. In Vitro and In Vivo Evidence

All four in vitro studies reported reduced proliferation or viability following berberine treatment [53,59,60,61]. All four also demonstrated reduced invasion or invasion-associated markers [53,59,60,61]. Two adenomyosis studies demonstrated induction of apoptosis and cell-cycle arrest [59,60], while one endometriosis study provided a mechanistic rescue experiment for miR-429 [61]. The consistency of the antiproliferative direction is therefore greater than the consistency of any individual molecular mechanism.

The two in vivo studies differed in disease, species, induction model, intervention, and administration route [62,63]. Purified berberine was studied only in tamoxifen-induced mouse adenomyosis and was administered intraperitoneally. The rat endometriosis study administered an oral multi-alkaloid fraction. Consequently, there is no disease-specific in vivo evidence for orally administered purified berberine in either endometriosis or adenomyosis.

#### 3.6.3. Purified Berberine and Multi-Alkaloid Preparations

Five of six studies evaluated purified berberine. All four purified-berberine in vitro studies reported suppression of proliferative or invasion-associated cellular phenotypes, and the purified-berberine mouse study reported reduced adenomyosis burden and hyperalgesia. Warowicka et al. [62] conducted the only study using a botanical multi-alkaloid fraction. Its findings were retained as indirect evidence because the contribution of berberine cannot be separated from that of the other identified alkaloids.

#### 3.6.4. Mechanistic Convergence

IL-6 and VEGF were each reduced in three adenomyosis studies [59,60,63]. MMP2 was reduced in the two adenomyosis cell studies and the endometriosis-oriented cell-line study [59,60,61], while NF-κB-related activity was reduced in two studies [60,63]. TLR4 expression was directly measured only in Zhu et al. [63]. Liu et al. [59] studied an LPS-stimulated system interpreted in relation to previously established TLR4 signaling but did not measure or perturb TLR4 in the reported experiments. No causal TLR4 intervention was performed in either study.

Cell-cycle arrest and apoptosis were reproduced in the two closely related adenomyosis EESC studies [59,60]. Non-coding RNA mechanisms differed between the endometriosis studies: miR-429 was supported by a functional overexpression/rescue experiment [61], whereas H19 was supported by parallel changes in expression and phenotype [53]. No specific molecular pathway was reproduced by causal perturbation in two independent research groups.

### 3.7. Mechanisms Not Investigated in the Included Studies

None of the six studies examined any component of the gut microbiota–barrier–estrogen axis—including microbial composition or diversity, short-chain fatty acids, bile acids, intestinal permeability, bacterial translocation, circulating LPS or β-glucuronidase activity. No study measured GLP-1, GLP-1 receptor signaling or other incretin-related endpoints. None determined estrogen and progesterone concentrations, estrogen and progesterone receptors, or direct steroid-receptor activity. Likewise, the H19 study did not measure estrogen signaling during berberine exposure. These effects have not been demonstrated directly in endometriosis/adenomyosis berberine studies. Their possible relevance is derived from other experimental and clinical settings and was evaluated separately in the mechanistic synthesis.

### 3.8. Safety, Exposure and Clinical Applicability

No included study provided clinical efficacy data. None measured berberine pharmacokinetics or linked experimental concentrations or doses to systemic or lesion-level exposure. The in vitro concentrations of purified berberine ranged from 20 to 200 µM, while the only purified-berberine animal study used intraperitoneal administration. The oral animal experiment used 1 g/kg of a mixed alkaloid fraction whose berberine content and systemic exposure were not quantified.

No study included a dedicated comprehensive toxicity assessment. Liu et al. [60] reported that normal endometrial stromal cells were less affected by berberine than eutopic or ectopic adenomyosis cells, but this comparison was designed as a viability experiment and does not establish systemic or reproductive safety. The animal studies did not provide systematic organ toxicity, clinical chemistry, or long-term reproductive safety data. No included study supports a clinically applicable dose of berberine for endometriosis or adenomyosis.

### 3.9. Risk of Bias, Reporting Limitations and Feasibility of Quantitative Synthesis

The four in vitro studies raised some concerns, mainly because biological replication was limited, masking was not reported, and the relationship between experimental berberine concentrations and in vivo exposure was generally unexplored. The Zhu et al. animal study had several unclear SYRCLE domains despite reported random allocation. Warowicka et al. was judged at high risk, principally because five animals allocated to the treatment group were excluded after model establishment had been assessed. Detailed study-level judgments are presented in Table A2.

The primary cell studies were based on three disease donors each [59,60]. Gu and Zhou used a single immortalized non-lesion-derived cell line [61]. Gao et al. used primary lesion-derived cells but did not experimentally manipulate H19 and contained inconsistent descriptions of exposure time points [53]. Warowicka et al. reported an unclear recurrence denominator, excluded five treated animals without lesions at the second laparotomy, and used a multi-component intervention, and most treatment-related multivariate metabolomic comparisons were not statistically validated [62]. Zhu et al. used individual animals for the principal phenotypic outcomes, while several molecular analyses were based on small, incompletely defined mixed-tissue samples [63]. These features precluded calculation of a common effect estimate and restricted subgroup comparisons to descriptive synthesis.

Experimental systems ranged from primary patient-derived cells and an immortalized cell line to tamoxifen-induced adenomyosis and surgically induced peritoneal endometriosis. Interventions differed in chemical composition, concentration, route, and duration, and most outcomes were reported as relative assay values or graphical data.

## 4. Discussion

### 4.1. Principal Findings

All four in vitro studies reported reduced stromal cell proliferation. Depending on the model, this was accompanied by cell-cycle arrest, apoptosis, reduced migration or invasion, and changes in inflammatory or angiogenesis-related mediators. In mice with adenomyosis, intraperitoneal berberine also reduced myometrial infiltration and thermal hyperalgesia [53,59,60,61,63]. Lower IL-6 and VEGF recurred in both patient-derived adenomyosis cells and the animal adenomyosis model, while MMP2 reduction was reported in adenomyosis-derived cells and the endometriosis-oriented cell-line study [59,60,61,63].

Three primary cell studies used material from only three patients each, and the two adenomyosis studies by Liu and colleagues came from the same group and used closely related experimental models. Gu and Zhou extended the evidence to endometriosis, although CRL-2615 cells were neither lesion-derived nor experimentally converted to an endometriotic phenotype. Gao et al. used primary stromal cells from ovarian endometriomas and found effects at 20 µM, below the 50–200 µM concentrations used in the adenomyosis studies, but again in only three donors [53,59,60,61].

The animal evidence adds lesion- and pain-related outcomes without resolving the question of oral purified berberine. Intraperitoneal berberine reduced myometrial infiltration and thermal hyperalgesia in tamoxifen-induced adenomyosis, whereas the oral endometriosis experiment used a Chelidonium majus fraction containing several protoberberine alkaloids and did not determine exposure to the individual constituents [62,63]. The first model establishes systemic activity of purified berberine; the second provides evidence for an orally administered botanical fraction whose effects cannot be assigned to berberine. Endometriosis and adenomyosis share local estrogen excess, progesterone resistance, inflammatory abnormalities and fibrotic remodeling, but differ in anatomical and cellular environment [4]. Findings from adenomyosis can therefore inform pathways potentially relevant to endometriosis without supplying the missing in vivo evidence for purified berberine in endometriosis. No clinical study has assessed pain, fertility, lesion response or recurrence in either disease.

### 4.2. Cellular, Inflammatory, and Nociceptive Effects

Across the cellular studies, reduced proliferation is the most recurrent effect, accompanied by inflammatory findings that converge most on LPS/TLR4/NF-κB signaling. Functional evidence for this axis in adenomyosis predates the berberine experiments: LPS increased stromal cell viability and inflammatory-invasive behavior, and pharmacological TLR4 blockade attenuated these effects [64]. In adenomyosis-derived stromal cells, berberine inhibited proliferation both after LPS stimulation and without added LPS, with cell-cycle arrest and increased apoptosis in both settings [59,60]. Both experimental settings also showed reduced expression of IL-6, IL-8, TGF-β, EGF, VEGF and MMP2 [59,60]. Without added LPS, increased cleaved caspase-3 accompanied apoptosis, while reduced NF-κB/p65 nuclear translocation and STAT3 phosphorylation accompanied the suppression of inflammatory and invasion-associated mediators [60]. In mice with adenomyosis, berberine treatment was associated with lower TLR4 expression in the uterus and dorsal root ganglia. The administration of berberine also lowered NF-κB/p65 phosphorylation and TNF-α, IL-6 and MCP-1 concentrations in the murine dorsal root ganglia [63].

The berberine literature shows that signaling responses vary with model, dose, and tissue. PI3K/AKT, AMPK, MAPK, NF-κB, mitochondrial function and autophagy have all been affected in a model-, dose- and tissue-dependent manner [41]. Apoptosis increased in proliferating adenomyosis-derived stromal cells, whereas oral berberine reduced apoptosis in acutely inflamed uterine tissue together with oxidative injury and TLR2/PI3K/AKT activity [48,59,60]. The observations are compatible with suppression of ectopic cell growth and protection of injured normal tissue, but do not support apoptosis induction as a general property of berberine.

Reductions in VEGF and MMPs fit the observed decrease in invasive behavior, but no included study measured vessel formation or lesion perfusion [59,60,61,63]. The animal pain data showed that improved hotplate and tail-flick responses coincided with lower TRPV1, COX-2 and inflammatory signaling in dorsal root ganglia, suggesting that changes in sensory pathways may contribute alongside the uterine response [63]. Pelvic pain in endometriosis and adenomyosis includes peripheral sensitization, lesion-associated innervation and altered central processing, so thermal withdrawal tests capture only part of the clinical phenotype and cannot distinguish an analgesic effect from pain reduction secondary to disease suppression [21,22,63].

### 4.3. Non-Coding RNAs and Steroid-Related Mechanisms

Overexpression of miR-429 attenuated the berberine-associated changes in proliferation, migration and invasion, giving this RNA a functional position within the observed cellular response, albeit in a cell line not directly derived from endometriotic lesions [61]. H19 was examined in a more disease-specific model: 20 µM berberine reduced H19 expression together with proliferation and invasion in primary ectopic stromal cells, but H19 was not experimentally restored or suppressed [53].

H19 also connects the berberine findings with steroid-responsive endometriosis biology. Increased H19 in ectopic cells has been linked with proliferation and invasion through miR-124-3p/ITGB3, while estradiol increases H19 in primary ectopic stromal cells and the estrogen-responsive H19/miR-216a-5p/ACTA2 axis has been associated with migration and invasion [65,66,67]. Its behavior in eutopic endometrium is different, since reduced H19 and altered H19/let-7/IGF1R signaling have been associated with impaired stromal cell growth and potentially receptivity [68]. A reduction of H19 in ectopic tissue may therefore have consequences distinct from the same change in eutopic endometrium, which becomes relevant if berberine is considered in women seeking pregnancy.

None of the six included studies measured estradiol, progesterone, ERα, ERβ, progesterone receptors, aromatase or receptor-dependent transcription. Outside endometriosis and adenomyosis, berberine inhibited both estradiol- and progesterone-stimulated proliferation in leiomyoma cells while normal myometrial cells were less affected at the same concentrations; direct ER or PR interaction was not examined [46]. The progesterone result cannot be transferred directly to endometriosis, where progestogenic signaling suppresses endometrial proliferation and therapeutic progestogens are central to treatment. Binding to GPER1 has been demonstrated in breast cancer cells together with changes in estrogen-dependent cellular responses, but this provides no information about the direction of GPER1-mediated effects in endometriotic tissue [69].

Steroid-responsive effects may also arise without a direct action on steroid receptors. In ovariectomized rats, oral berberine changed gut microbial composition and increased production of equol, a daidzein metabolite with preferential ERβ affinity; the associated phenotype was markedly weakened in germ-free animals and partly transferred by fecal microbiota, without restoration of circulating estradiol or progesterone [47]. In endometriosis, this is difficult to interpret because ERβ has been linked experimentally with lesion survival, inflammatory signaling and resistance to apoptosis [70,71]. Clinical co-administration of berberine and progesterone has been described in PCOS, but the different endocrine setting provides no evidence on progestogen responsiveness or reproductive safety in endometriosis [72].

### 4.4. Gut Microbiota and Microbial Metabolites

An intestinal component to berberine action is pharmacologically plausible because conventional oral berberine is poorly absorbed and extensively metabolized, producing gastrointestinal exposure very different from systemic exposure [41]. Intestinal bacterial nitroreductases convert berberine to dihydroberberine, which showed roughly fivefold greater intestinal absorption in animal experiments; antibiotic depletion reduced this conversion, circulating exposure, and the metabolic response [43]. Berberine also changes the gut microbiota in humans, although no reproducible taxonomic “healthy microbiome” pattern has emerged across the seven randomized trials summarized by Candrea et al. [73]. In an oral berberine mouse model, treatment altered microbial composition, restored reduced butyrate, improved intestinal permeability and tight-junction expression, and lowered bacterial translocation and plasma LPS; microbiota depletion attenuated much of this response [74]. These experiments support a microbiota-dependent gut–barrier–LPS pathway of oral berberine.

In experimental endometriosis, antibiotic treatment and metronidazole reduced lesion growth and inflammation, while fecal transfer from mice with endometriosis restored lesion growth [75]. Microbial metabolites themselves can modify disease behavior: *n*-butyrate inhibited human endometriotic cell survival and reduced experimental lesions, whereas quinic acid promoted endometriotic epithelial cell survival and lesion growth [76,77]. These functional data provide stronger support for microbial involvement than the less consistent human taxonomic findings [50,78].

Human studies have identified differences in gut, uterine, and reproductive-tract microbiota, but no single bacterial composition has emerged that berberine would simply need to “normalize.” A large gut metagenomic study found no significant differences in species, diversity, functional pathways, or estrobolome-related enzymes after correction for multiple testing [79], and analysis of 266 uterine samples found no individual endometriosis-associated taxon surviving FDR correction, with additional variation according to menstrual-cycle phase [80]. In adenomyosis, altered intestinal microbial structure has been reported in treatment-naïve patients, while reproductive-tract studies have identified disease- and symptom-associated microbial differences [81,82]. An endometrial multi-omics study found both shared and disease-specific microbial–metabolic alterations in endometriosis and adenomyosis, but each also showed distinct microbial and metabolic profiles [83].

Increased intestinal permeability can increase exposure to microbial products, such as LPS, while TLR4 activation promotes proliferation and inflammatory-invasive behavior in adenomyosis stromal cells [64]. The same LPS/TLR4 axis appears in the included berberine studies: LPS-induced proliferation and inflammation were suppressed in adenomyosis stromal cells, and TLR4/NF-κB-associated activity was reduced in the adenomyosis mouse model [59,63]. Oral berberine can also lower intestinal permeability and circulating LPS in a microbiota-dependent setting [74]. These components have therefore been demonstrated separately, but no endometriosis or adenomyosis study has yet connected oral berberine-induced microbial or barrier changes with the pelvic TLR4/NF-κB response.

Bacterial β-glucuronidases provide a second, less direct connection by altering enterohepatic estrogen recycling, while microbiota-dependent effects of berberine on estrogen-responsive biology have been demonstrated experimentally [47]. None of the included studies measured the estrobolome, circulating estrogen turnover, or microbiota-dependent steroid signaling, and the large human endometriosis metagenomic study found no difference in estrobolome-related enzyme abundance [79]. The microbiota–barrier–LPS/TLR4 pathway is therefore currently better connected to the disease-specific berberine evidence than a microbiota-mediated effect on estrogen metabolism.

### 4.5. GLP-1-Related Mechanisms

Berberine increased endogenous GLP-1 secretion in human enteroendocrine cells through TAS2R38 and PLC signaling, and TAS2R38 knockdown attenuated the response [49]. This places the observed action upstream of GLP1R and provides no basis for describing berberine as a GLP1R agonist. In endometriosis, lower peritoneal-fluid GLP-1 was reported in 54 affected women compared with 30 women with uterine myomas, together with a positive association between GLP-1 and macrophage CD86 expression [51]. The cross-sectional design leaves the direction and biological significance of this association unresolved, and detection of GLP1R does not demonstrate receptor-dependent disease activity. None of the six included berberine studies measured GLP-1 or GLP1R. Microbial metabolites and bile acids provide possible links between intestinal berberine exposure and GLP-1 secretion, but no experiment has connected this sequence with endometriosis or adenomyosis.

**Figure 2:**
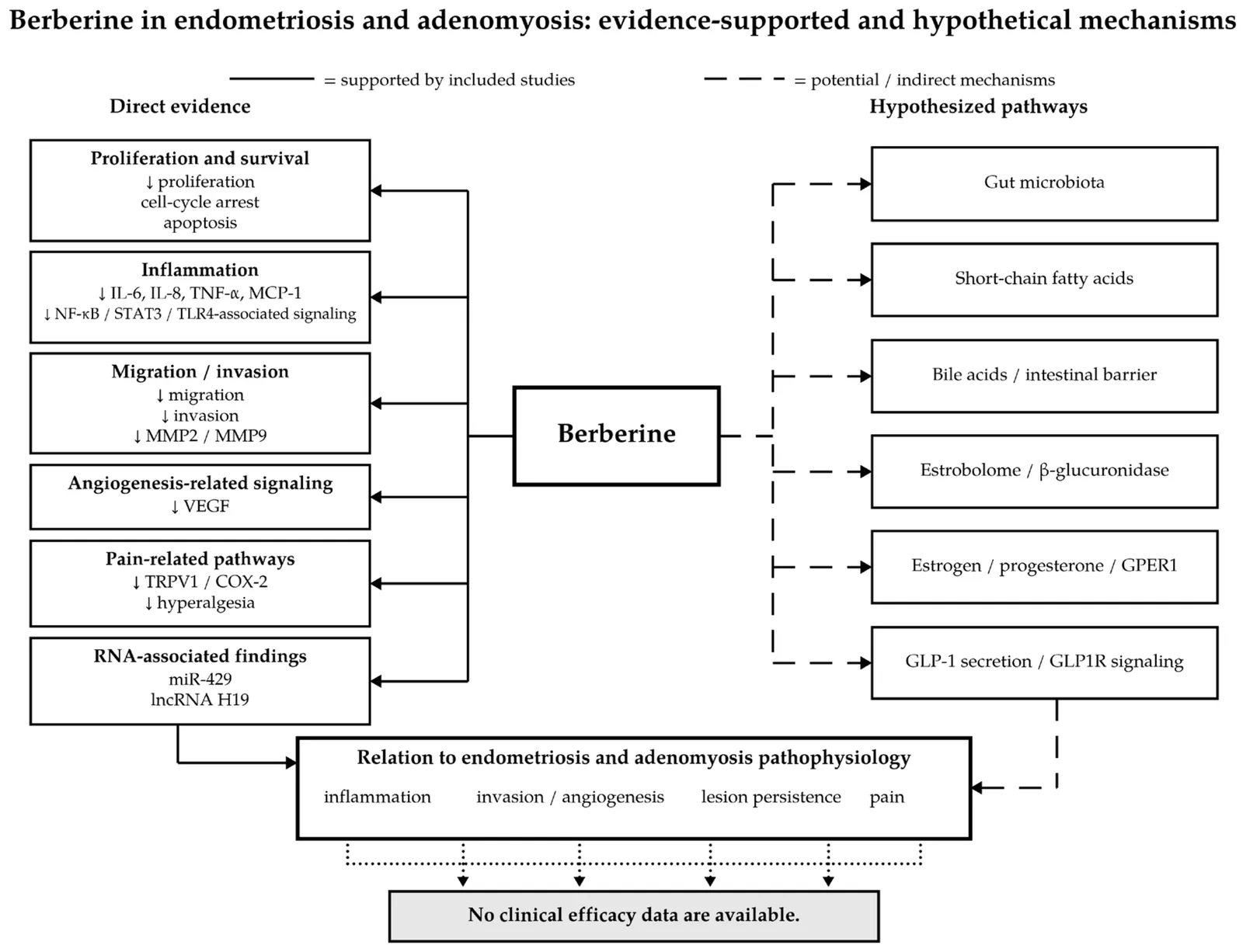
Observed and hypothetical effects of berberine relevant to endometriosis and adenomyosis.

The disease-related effects observed in the included studies and the pathways that remain hypothetical in endometriosis and adenomyosis are summarized in Figure 2.

### 4.6. Pharmacokinetics, Dose, and Safety

The 20–200 µM concentrations used in vitro remain difficult to interpret clinically because none of the cellular studies measured intracellular berberine or demonstrated comparable tissue exposure after oral administration [53,59,60,61]. Oral bioavailability of the parent compound is below 1% in conventional formulations because of limited absorption and extensive intestinal and hepatic metabolism [41].

Purified berberine was effective after intraperitoneal administration at 5–20 mg/kg/day in adenomyosis, while oral treatment in endometriosis involved 1 g/kg of a multi-alkaloid fraction with undefined berberine exposure [62,63]. No study has related an oral dose of purified berberine to plasma and lesion concentrations and a disease outcome.

Clinical experience from other indications mainly identifies gastrointestinal adverse effects, including constipation and diarrhea, and the quality of the safety evidence remains variable [42]. None of the disease-specific studies examined implantation, ovarian function, early pregnancy exposure or interactions with hormonal therapy. These gaps are directly relevant to endometriosis and adenomyosis, where long-term progestogen use and attempts to conceive are common clinical settings.

### 4.7. Acceptance of Hormonal Treatment and Demand for Alternatives

Social media has broadened access to peer experience outside the clinical consultation and has contributed to a shift in risk perception regarding hormonal contraception, with greater attention to effects on mood, sexuality, well-being, and bodily autonomy [84,85]. In an online survey conducted via social media in Austria, Germany, and Switzerland and including 1420 respondents with self-reported endometriosis, 66.1% were not satisfied with the current endocrine treatments, and 63.5% described their attitude towards these drugs as negative. A negative perception of these drugs and dissatisfaction were particularly frequent, at approximately 70%, among respondents aged ≤30 years, living in large cities, or holding an academic degree. The most frequently reported reasons were side effects in general (85.5%), changes in libido (67.5%), hormonal cycle disruptions (65.9%), and insufficient efficacy in alleviating symptoms (38.2%) [86]. In a 2026 real-world patient-reported study of dienogest, a commonly used first-line progestin for endometriosis, fatigue, decreased libido, and mood disturbances were reported by 63.0%, 58.3%, and 50.7% of 146 participants, respectively, and 40.4% had discontinued treatment, mainly because of adverse events [87]. These negative attitudes and experiences are concordant with those reported for hormonal contraceptive methods. In Germany, pill use among women with contraceptive needs decreased from 45.7% in 2012 to 25.3% in 2024 [88]. A systematic review of 42 studies found that reasons for rejecting hormonal contraception included physical and mental adverse effects, effects on sexuality, concerns about future fertility, and the perceived delegitimization of side effects by healthcare professionals [89]. A recent synthesis of 50 qualitative studies similarly found that such concerns often reflect lived and contextual experience and should not be reduced to misinformation [90]. This wider access to patient experience can address gaps in conventional health communication, while the same platforms can amplify selective or inaccurate claims and therefore influence health behavior in either direction [85].

### 4.8. Social Media and Evidence Communication

At the time of our assessment, more than 63,000 Instagram posts carried the hashtag *#berberine*, while the disease-specific literature consisted of six preclinical studies and no clinical trials. The hashtag count measures general visibility and not endometriosis-related use, but endometriosis-oriented websites already discuss berberine in relation to inflammation, estrogen metabolism, microbiota and fertility, and some provide specific oral regimens [36,37,38]. In regard to endometriosis, social-media analyses show extensive exchange about symptoms, diagnostic experiences, psychological burden and coping, often in simple language, but of unproven scientific quality [91,92]. Among 515 Instagram posts, only four of eleven educational categories contained at least 50% evidence-based claims [35]. A later systematic review found that most evaluated endometriosis content was produced by non-healthcare professionals, with misinformation concentrated particularly around treatments and complementary approaches [92]. An analysis of Instagram during Endometriosis Awareness Month 2025 showed most Instagram content lacked evidentiary depth. In particular, 88% of posts did not sufficiently inform about treatment options and 92% did not include dietary aspects. Healthcare professional authorship and a natural tone were associated with more likes [93]. We assume that interest in berberine has been amplified by the social-media “hype” around GLP-1-related medications (in August 2026 there were one million posts marked with the hashtag *#glp1* on Instagram, 800,000 on TikTok), given berberine’s reported ability to stimulate GLP-1 secretion in experimental settings [49]. A content analysis of GLP-1-related reporting on TikTok showed that 98% was produced by individual users or influencers, popularity metrics (views and likes) did not correlate with information quality, and a significant proportion of the content contained risky or unverified information, especially regarding adverse effects and usage safety [94]. In gynecologic contexts (hysteroscopy-related content), inaccuracies were identified in 36.4% of Instagram videos and 42.9% of TikTok videos, and 8.5% on YouTube [95]. There is no reason to expect that social-media reporting on berberine or endometriosis will be exempt from premature efficacy claims or currently unsupported recommendations.

### 4.9. Strengths and Limitations

The review separates findings obtained directly in endometriosis or adenomyosis from hypotheses derived from adjacent research and distinguishes experimentally tested pathway involvement, such as the miR-429 rescue experiment, from molecular associations alone [53,61]. Placing six preclinical studies beside tens of thousands of social media posts and website entries exposes—and tries to fill—a gap between disease-specific evidence and the confidence of current public discussion [35,92].

The small evidence base remains the principal limitation. Several findings come from related experiments with very few donors. One endometriosis study used an immortalized HESC line. The in vivo studies differ in disease, intervention and route of administration [61,62,63]. These differences precluded quantitative synthesis. Context studies were used only where they addressed a specific link to the disease-specific findings. Pharmacological similarities observed in metabolic, cardiovascular, malignant, or neurological models cannot be translated into treatment recommendations for endometriosis or adenomyosis.

### 4.10. Research Priorities and Clinical Implications

An oral purified-berberine model with concurrent pharmacokinetic measurements would help relate oral dosing to tissue exposure. Parent compound and major metabolites need to be measured in plasma, intestine, uterus and lesions together with lesion and pain outcomes, allowing subsequent cellular experiments to use concentrations that can actually be reached in vivo [41,43]. Without such data, experiments at 20–200 µM establish pharmacological activity in culture but cannot determine its contribution after oral treatment.

miR-429 can be examined in primary lesion-derived cells, H19 requires functional manipulation during berberine exposure, and TLR4 dependence can be tested directly because functional TLR4 data already exist in adenomyosis [53,61,64]. Experiments combining berberine with estradiol and clinically used progestogens could determine its effects on ER-, PR- and GPER1-dependent responses and clarify whether H19 regulation persists under hormonal stimulation [46,67,69].

Testing an intestinal route requires gut and pelvic endpoints within the same experiment. Oral berberine can be studied together with microbial metabolites, barrier permeability or circulating LPS, pelvic inflammatory signaling and disease outcomes, followed by microbial depletion or transfer where appropriate [47,75]. GLP-1 receptor-level experiments could be combined with measurements of circulating or peritoneal GLP-1 after oral berberine [49,51].

Any clinical efficacy study will require a defensible oral dose and better reproductive safety data. Current evidence provides neither a dose recommendation nor clinical data on pelvic pain, fertility, lesion regression, recurrence or disease progression. Specific dosing recommendations already circulating online therefore precede the pharmacokinetic and clinical evidence needed to support them.

## 5. Conclusions

Six preclinical studies show that berberine can affect biological processes relevant to endometriosis and adenomyosis, with reduced stromal cell proliferation as the most consistently reproduced finding. Evidence linking individual pathways to these effects is less complete: miR-429 has been tested functionally, whereas H19, TLR4/NF-κB, STAT3 and several other molecular findings remain associative or only partly tested. Gut microbiota, steroid-responsive signaling and GLP-1 connect berberine pharmacology with established aspects of endometriosis or adenomyosis biology, but none has yet been demonstrated as a disease-specific route of berberine action. The low systemic availability of oral berberine and the high concentrations used in vitro leave tissue exposure unresolved, while substantial intestinal exposure makes gut-dependent effects particularly relevant for future investigation. No clinical efficacy data, disease-specific oral dose or adequate reproductive safety data are available. These data justify further experimental work but do not support berberine as an evidence-based treatment for endometriosis or adenomyosis.

## Figures and Tables

**Table 1:** Characteristics and main findings of the included studies.

Study	Disease and Model	Berberine Intervention	Main Findings	Mechanistic Findings	Main Limitations Relevant to Interpretation
Liu et al., 2017 [59]	Adenomyosis; primary EESCs from 3 patients; LPS-stimulated model	Purified BBR 50, 100, 200 µM; 24–72 h; mechanistic experiments mainly 200 µM for 48 h	Dose- and time-dependent inhibition of LPS-associated proliferation; G0/G1 arrest; increased apoptosis	↓ IL-6, IL-8, TGF-β, EGF, VEGF, MMP2	Three donors; TLR4 was not measured or experimentally perturbed despite the LPS/TLR4 interpretation; no normal-cell toxicity control; no exposure justification
Liu et al., 2018 [60]	Adenomyosis; primary NESCs, EuESCs and EESCs; 3 healthy and 3 adenomyosis donors	Purified BBR 50–200 µM; 24–72 h	Reduced viability of EuESCs and EESCs; comparatively small effect on NESCs; G1 arrest and apoptosis	↑ cleaved caspase-3; ↓ IL-6, IL-8, TGF-β, EGF, VEGF, MMP2; reduced NF-κB/p65 nuclear translocation and STAT3 activation	Three disease donors; safety data limited to normal-cell viability; high concentrations without pharmacokinetic justification
Gu and Zhou, 2021 [61]	Endometriosis-oriented in vitro study; immortalized HESC CRL-2615	Purified BBR 20–80 µM; 80 µM used for most experiments	Reduced viability, colony formation, EdU incorporation, migration and invasion	↓ Ki-67, PCNA, MMP2/MMP9 and miR-429; miR-429 overexpression reversed several BBR effects	Cell line was neither lesion-derived nor given an induced endometriosis phenotype; no normal-cell comparator; experimental unit incompletely reported
Warowicka et al., 2021 [62]	Endometriosis; surgically induced peritoneal autotransplantation model in rats	*C. majus* protoberberine-rich fraction, 1 g/kg orally for 14 days	Authors reported recurrent lesions in 12.5% of treated animals versus 61% of model controls (*p* = 0.02); stromal atrophy in recurrent treated lesions	Serum metabolomic differences involving energy-related metabolites were reported	Multi-alkaloid intervention prevents attribution to BBR; 5/21 treated animals without lesions at second laparotomy were excluded; recurrence denominator is internally inconsistent; most OPLS-DA comparisons did not pass CV-ANOVA validation
Zhu et al., 2022 [63]	Adenomyosis; tamoxifen-induced neonatal ICR mouse model; n = 12 per group for uterine weight, infiltration scoring and thermal nociception outcomes.	Purified BBR, 98% HPLC purity; 5, 10, 20 mg/kg/day i.p. for 3 weeks	Reduced uterine weight and myometrial infiltration at higher doses; dose-related improvement in hotplate and tail-flick latency	↓ TRPV1, COX-2, VEGF, OTR; ↓ DRG NF-κB/p65 activation, TNF-α, IL-6 and MCP-1; ↓ TLR4 in DRG and uterus	Intraperitoneal route; many molecular analyses used small and incompletely defined pooled/mixed-tissue samples; no dedicated toxicity assessment; TLR4 dependence was not experimentally tested
Gao et al., 2025 [53]	Endometriosis; primary ecESCs from ovarian endometriotic cysts of 3 patients	Purified BBR 20 µM	Reduced proliferation and Transwell invasion	↓ lncRNA H19; H19 changes correlated with proliferation and invasion	Three donors and three repeat wells; no H19 knockdown/rescue experiment within the study; inconsistently reported treatment time points; no dose-response assessment for BBR

Abbreviations: BBR, berberine; DRG, dorsal root ganglion; EESC/ecESC, ectopic endometrial stromal cell; EuESC, eutopic endometrial stromal cell; HESC, human endometrial stromal cell; LPS, lipopolysaccharide; NESC, normal endometrial stromal cell.

## Data Availability

No new data were created or analyzed in this study. Data sharing is not applicable to this article.

## Supplementary Materials

The following supporting information can be downloaded at: https://www.mdpi.com/article/10.3390/nu18213647/s1, Table S1: PRISMA 2020 Checklist.

## Abbreviations

ACTA2, actin alpha 2, smooth muscle; AMPK, AMP-activated protein kinase; BBR, berberine; CCK-8, Cell Counting Kit-8; CD86, cluster of differentiation 86; COX-2, cyclooxygenase-2; CV-ANOVA, cross-validated analysis of variance; DRG, dorsal root ganglion; EESC/ecESC, ectopic endometrial stromal cell; EdU, 5-ethynyl-2′-deoxyuridine; EGF, epidermal growth factor; ERα/ERβ, estrogen receptor alpha/beta; EuESC, eutopic endometrial stromal cell; FDR, false discovery rate; FODMAP, fermentable oligosaccharides, disaccharides, monosaccharides and polyols; GLP-1, glucagon-like peptide-1; GLP1R, glucagon-like peptide-1 receptor; GPER1, G protein-coupled estrogen receptor 1; HESC, human endometrial stromal cell; HPLC, high-performance liquid chromatography; IGF1R, insulin-like growth factor 1 receptor; IL, interleukin; ITGB3, integrin beta-3; lncRNA, long non-coding RNA; LPS, lipopolysaccharide; MAPK, mitogen-activated protein kinase; MCP-1, monocyte chemoattractant protein-1; miR/miRNA, microRNA; MMP, matrix metalloproteinase; NESC, normal endometrial stromal cell; NF-κB, nuclear factor kappa B; NMR, nuclear magnetic resonance; OPLS-DA, orthogonal partial least squares discriminant analysis; OSF, Open Science Framework; OTR, oxytocin receptor; PCNA, proliferating cell nuclear antigen; PCOS, polycystic ovary syndrome; PI3K/AKT, phosphoinositide 3-kinase/protein kinase B; PLC, phospholipase C; PR, progesterone receptor; PRISMA, Preferred Reporting Items for Systematic Reviews and Meta-Analyses; SCFA, short-chain fatty acid; STAT3, signal transducer and activator of transcription 3; SYRCLE, Systematic Review Centre for Laboratory Animal Experimentation; TAS2R38, taste receptor type 2 member 38; TGF-β, transforming growth factor beta; TLR2/TLR4, Toll-like receptor 2/4; TNF-α, tumor necrosis factor alpha; TRPV1, transient receptor potential vanilloid 1; VEGF, vascular endothelial growth factor.

## Appendix A

**Table A1:** Structured qualitative assessment of confidence in the synthesized findings.

Domain	Evidence Assessed	Estimated Confidence
Risk of bias and precision	Three primary-cell studies [53,59,60] each used three disease donors. Masking was not reported. The animal studies [62,63] had unclear domains or high risk of bias.	Confidence is limited. Consistent directions cannot exclude systematic error or establish precise effect sizes.
Consistency and independence	All four cellular studies [53,59,60,61] reported reduced proliferation or viability. IL-6, VEGF and MMP2 recurred across reports [59,60,61,63].	Cellular direction is more recurrent than any causal pathway.
Directness	Three cellular studies used patient-derived material [53,59,60]. One used a non-lesion-derived cell line [61]. Purified berberine was tested in vivo only in adenomyosis [63]; the endometriosis animal experiment used a multi-alkaloid fraction [62].	Disease and intervention directness vary. Fraction effects cannot be assigned specifically to berberine.
Biological plausibility	Growth, inflammatory and invasion-associated findings concern relevant disease processes [53,59,60,61,63]. One miR-429 rescue experiment [61] tested functional involvement; most other pathways were measured as associations [53,59,60,63].	Plausibility supports testable hypotheses. It does not establish causal mediation or validate gut, steroid or GLP-1 mechanisms in these diseases.
Dose and exposure relevance	Cellular concentrations were 20–200 µM [53,59,60,61]. Purified berberine was given intraperitoneally in the animal study [63]. No study measured plasma or lesion concentrations [53,59,60,61,62,63].	The relevance of experimental exposure to an oral human regimen remains unresolved.
Translational relevance	No eligible clinical study, oral purified-berberine disease model, or dedicated reproductive safety assessment was identified in the included evidence [53,59,60,61,62,63].	Clinical efficacy, an effective oral dose and reproductive safety are unestablished; this is absence of direct evidence, not a graded estimate of no effect.

**Table A2:** Risk of bias and quality assessment of included studies.

Study	Design/Model	Tool	Overall Judgment	Main Reason
Liu et al., 2017 [59]	Primary ectopic stromal cells from adenomyosis; LPS-stimulated model	Structured in vitro appraisal	Some concerns	Disease-derived and characterized cells with dose-response experiments and repeated assays, but only three donors, no reported masking, and high berberine concentrations (50–200 µM) without pharmacokinetic justification. TLR4 dependence was inferred from the LPS model but not directly tested during berberine exposure.
Liu et al., 2018 [60]	Primary normal, eutopic, and ectopic endometrial stromal cells	Structured in vitro appraisal	Some concerns	Comparative primary-cell design and multiple cellular and molecular endpoints, but very small biological sample (three adenomyosis patients and three controls), no reported masking, 100–200 µM berberine exposure without pharmacokinetic justification, and no functional perturbation of NF-κB or STAT3.
Gu and Zhou, 2021 [61]	Immortalized CRL-2615 endometrial stromal cell line	Structured in vitro appraisal	Some concerns	Multiple independent cellular assays and a functional miR-429 overexpression experiment strengthen internal inference. The cell line (HESC) was not directly lesion-derived and had no experimentally established endometriotic phenotype; only one cell line was used and masking was not reported, and the principal 80 µM exposure lacked pharmacokinetic justification.
Warowicka et al., 2021 [62]	Surgically induced rat endometriosis; oral protoberberine-rich *Chelidonium majus* fraction	SYRCLE	High risk	Random allocation was reported, but sequence generation, allocation concealment, blinding, and random outcome assessment were insufficiently described. Five of 21 animals allocated to the treatment group were excluded after the second laparotomy because endometriosis had not developed. The multi-alkaloid intervention additionally prevents attribution of the findings specifically to berberine.
Zhu et al., 2022 [63]	Tamoxifen-induced neonatal mouse adenomyosis; purified berberine i.p.	SYRCLE	Some concerns/unclear	Mice were randomly assigned to model/control groups and adenomyosis mice were randomly divided into treatment groups; dose-response, histological, molecular and behavioral endpoints were reported. Sequence generation, allocation concealment and assessor blinding were not described, which is particularly relevant to hotplate, tail-flick and infiltration scoring.
Gao et al., 2025 [53]	Primary ectopic stromal cells from ovarian endometriomas	Structured in vitro appraisal	Some concerns	Lesion-derived primary cells and direct proliferation/invasion assays improve disease relevance, and the 20 µM concentration was lower than in the earlier cellular studies. Biological replication was limited to three donors, masking was not reported, only one berberine concentration was examined and H19 was measured but not experimentally manipulated.

Abbreviations: SYRCLE, Systematic Review Centre for Laboratory Animal Experimentation. For in vitro studies, methodological limitations were assessed using the predefined structured appraisal described in Section 2.6. “Some concerns” in these studies denotes limitations in experimental design, reporting, biological replication, or model applicability and is not a category derived from a validated in vitro risk-of-bias instrument. For animal studies, the overall judgment summarizes the relevant SYRCLE domains.
